# DUSP22 Ameliorates Endothelial-to-Mesenchymal Transition in HUVECs through Smad2/3 and MAPK Signaling Pathways

**DOI:** 10.1155/2024/5583961

**Published:** 2024-03-08

**Authors:** Lu Chen, Hongyu Su, Zekai Tao, Cui Liang, Zhongzhao Liu, Yiming Dong, Peipei Zheng, Yuan Liu

**Affiliations:** Department of Cardiology, The First Affiliated Hospital of Zhengzhou University, Zhengzhou, China

## Abstract

Endothelial-to-mesenchymal transition (EndMT) is the process by which endothelial cells lose their endothelial properties and acquire mesenchymal characteristics. Dual-specific protein phosphatase 22 (DUSP22) inactivates various protein kinases and transcription factors by dephosphorylating serine/threonine residues: hence, it plays a key role in many diseases. The aim of this study was to explore the functional role of DUSP22 in EndMT. In the transforming growth factor-*β*-induced EndMT model in human umbilical vein endothelial cells (HUVECs), we observed a downregulation of DUSP22 expression. This DUSP22 deficiency could aggravate EndMT. Conversely, the overexpression of DUSP22 could ameliorate EndMT. We used signaling pathway inhibitors to verify our results and found that DUSP22 could regulate EndMT through the smad2/3 and the mitogen-activated protein kinase (MAPK) signaling pathways. In summary, DUSP22 ameliorates EndMT in HUVECs in vitro through the smad2/3 and MAPK signaling pathways.

## 1. Introduction

Myocardial fibrosis is the result of myocardial fiber persistence and/or repeated myocardial ischemia and hypoxia caused by moderate and severe coronary artery stenosis, leading to chronic ischemic heart disease that gradually develops into heart failure [1]. At present, it is believed that myocardial fibrosis is a complex pathological process, involving the renin-angiotensin aldosterone system, immune system, and various cytokines; inflammation, cell apoptosis, and cell signal regulation are all related to its occurrence [2].

Endothelial-to-mesenchymal transition (EndMT) belongs to a special type of epithelial-to-mesenchymal transition (EMT) [3]. It is a process in which endothelial cells transform into mesenchymal cells activated by various stimuli. During this process, endothelial cells gradually lose their shape and function and acquire the phenotypic characteristics of mesenchymal cells such as proliferation, migration, and collagen synthesis [4]. Endothelial cells transform from a “cobblestone” monolayer phenotype to spindle-shaped cells; lose expression of characteristic hallmark proteins including vascular endothelial cadherin (VE-cadherin), CD31/platelet-endothelial cell adhesion molecule-1 (CD31/PECAM-1), von Willebrand factor (vWF), and vascular endothelial growth factor (VEGF); and gain expression of characteristic biomarkers of mesenchymal cells including *α*-smooth muscle protein (*α*-SMA), fibroblast-specific protein-1 (FSP1), vimentin, fibronectin, connective tissue growth factor (CTGF), N-cadherin, and collagen I/III [5]. EndMT also plays a key role in cardiovascular diseases, such as cardiac fibrosis [6], atherosclerosis [7, 8], atrial fibrillation [9], myocardial infarction [10], cardiac remolding [11], and heart failure [12, 13].

The dual-specific protein phosphatase (DUSP) family belongs to a subclass of the protein tyrosine phosphatase (PTP) family [14] that inactivates mitogen-activated protein kinase (MAPK) and other tyrosine kinases through dephosphorylation [15, 16]. DUSPs limit the function of target kinases through dephosphorylation of target kinases to prevent abnormal activation [17, 18]. Many DUSPs have become potential therapeutic targets in many diseases, such as skin cancer [19, 20], non-small-cell lung cancer [21], lymphoma [22], systemic lupus erythematosus [23], and lymphocytic leukemia [24]. Numerous studies have shown that DUSPs are crucial to the regulation of MAPK activity and therefore impact complex regulatory switches [25–29]. Gene expression and phosphatase activity of DUSPs are affected by gene transcription, protein modification, and protein stability [15].

DUSP22 is an atypical PTP consisting of 184 amino acid residues that is widely expressed in a variety of human tissues. DUSP22, also known as c-JUN N-terminal kinase (JNK) pathway-associated phosphatase (JKAP), dephosphorylates P38/mitogen-activated protein kinase (MAPK) [30], JNK/MAPK [31], estrogen receptor (ER) [32], focal adhesion kinases (FAK) [33], lymphocyte-specific protein tyrosine kinase [34], and signal transducer and activator of transcription 3 (STAT3) [35] and regulates T-cell receptor signaling [36]. Hence, DUSP22 plays a key role in controlling multiple signaling pathways. Some studies have shown that the above-mentioned pathways can also participate in the process of EndMT. However, there are no reports about the role of DUSP22 in cardiovascular diseases. It would be useful to determine whether DUSP22 can regulate EndMT—this may lead to new therapeutic strategies for cardiovascular diseases such as cardiac hypertrophy, myofibrosis, cardiac remodeling, and heart failure.

## 2. Methods

### 2.1. Cell Culture

Human umbilical vein endothelial cells (HUVECs) (purchased from Zhongqiao Xinzhou, China) were cultured in a 5% CO_2_ incubator at 37°C. HUVEC complete medium components include endothelial cell medium, 5% fetal bovine serum, 1% penicillin/streptomycin solution, and 1% endothelial cell growth supplement (purchased from ScienCell, USA). HUVEC starvation medium components include endothelial cell medium, 1% fetal bovine serum, 0.2% penicillin/streptomycin solution, and 0.2% endothelial cell growth supplement. When the cell density reached 80%-90% confluence, we used 0.25% EDTA-trypsin (purchased from Gibco, USA) for digestion and passage of cells. We established an *in vitro* EndMT model by incubating HUVECs for 48 h in HUVEC starvation medium containing 10 ng/ml transforming growth factor-*β* (TGF-*β* purchased from PeproTech, USA). We chose smad2/3 small molecule inhibitor ITD-1, JNK inhibitor SP600125, and extracellular signal-regulated kinase (ERK) inhibitor ravoxertinib (purchased from Selleck, USA) to verify the DUSP22 signaling pathway.

### 2.2. Quantitative Real-Time Polymerase Chain Reaction (RT-PCR)

Total RNA was extracted from HUVECs. RNA (2 *μ*g per sample) was reverse-transcribed into cDNA using TB Green® Premix Ex Taq™ II kit (purchased from Takara, USA). We then used a QuantStudio 3 kit (Thermo Fisher, USA) to perform PCR analysis using PrimeScript™ RT reagent kit with gDNA Eraser which was purchased from Takara (USA). GAPDH was used as the internal reference gene. The oligonucleotide primer sequences used are shown in Table 1.

### 2.3. Western Blot Analysis

HUVECs were lysed in radioimmunoprecipitation assay lysis buffer (purchased from Solarbio, China). Protein samples (20 *μ*g) were separated by sodium dodecyl sulfate polyacrylamide gel electrophoresis and transferred to a polyvinylidene difluoride membrane (Millipore, China), which was then incubated with different primary antibodies, including CD31 (ab9498), vimentin (ab92547), DUSP22 (ab70124), *α*-SMA (ab124964), E-cadherin (ab40772), VE-cadherin (ab33168), smad2 (ab40855), smad3 (ab40854), smad2 (phospho S467) (ab280888), smad3 (phosphoS423+S425) (ab52903), ERK1+ERK2 (ab184699), ERK1 (phospho T202)+ERK2 (phospho T185) (ab201015), JNK1+JNK2+JNK3 (ab179461), and JNK1+JNK2+JNK3 (phosphoT183+T183+T221) (ab124956) (purchased from Abcam, USA) and GAPDH (2118S) and N-cadherin (13116S) (purchased from Cell Signaling Technology, USA). The blots were developed with enhanced chemiluminescence reagents (purchased from Millipore, USA) and captured by chemiluminescence imager (AI680). GAPDH served as an internal reference protein.

### 2.4. Virus Transfection

HUVECs with good growth status at passages 2-5 were selected for experiments. On the first day, we used HUVEC complete medium to prepare a cell suspension with a density of (3 − 5) × 104/ml, added 2 ml/well cell suspension into 6-well plates, and cultivated the cells in a cell incubator at 37°C for 24 hours until the cell confluency was 20%-30%. In the next day, we replaced culture medium with transfection medium (1 ml per well) consisting of 40 *μ*l 25X transfection reagent and a specific volume of viral suspension (purchased from GeneChem, China) corresponding to the final viral titer required in a final complete medium volume of 1 ml. On the third and fourth day, after culturing for 24 hours, we replaced transfection medium with fresh complete medium and continued culturing the cells. On the fifth day, at 72 h post-transfection, we determined the transfection efficiency via a fluorescent microscope.

### 2.5. Cell Viability Assay Using the Cell Counting Kit-8 (CCK-8)

HUVECs with good growth status at passages 2-5 were selected for experiments. A 96-well plate was used for a cell viability assay via the CCK-8 kit (purchased from Dojindo Laboratory, Japan). We used HUVEC complete medium to prepare a cell suspension with a density of (3 − 5) × 104/ml, plated 100 *μ*l cell suspension in each well, and placed the 96-well plate in a 37°C cell culture incubator for 24 h. Next, the mixed virus culture solution of sh-NC, sh-DUSP22, OE^NC^, and OE^DUSP22^ was added to the 96-well plate to infect the cells. We also added 100 *μ*l serum-free medium (containing 10 *μ*l CCK-8 solution) to each well at different time points (0 h, 24 h, 48 h, 72 h, and 96 h) after transfection, measuring the absorbance value at 450 nm wavelength under a microplate reader after placing these plates in a cell culture incubator at 37°C for 2-4 h.

### 2.6. Cell Migration Assay (Cell Scratch Test)

We measured the cell migration ability of HUVECs by using a wound healing or cell scratch assay. Before inoculating cells on the culture plate, we used a black marker pen to draw horizontal lines on the back of the 6-well plate with a ruler, drawing three lines per well. The sh-NC, sh-DUSP22, OE^NC^, and OE^DUSP22^ group cells were virally transfected, harvested, and plated into 6-well plates. After cells had reached 80%-90% confluence, they were incubated with HUVEC starvation medium for 6 hours. The knockdown group was divided into the sh-NC, sh-NC+TGF-*β*, sh-DUSP22, and sh-DUSP22+TGF-*β* groups. The cells in the overexpression group were divided into the OE^NC^, OE^NC^+TGF-*β*, OE^DUSP22^, and OE^DUSP22^+TGF-*β* groups. We used a fine pipette tip to make cell scratches perpendicular to the well and ensured that the width of each scratch was consistent. After changing the medium, we acquired micrographs with an inverted fluorescence microscope at 0 h and 24 h incubation in a 37°C incubator. We analyzed the micrographs to determine cell mobility in the various experimental groups.

### 2.7. Transwell Cell Migration Assay

We precooled the pipette tips, 24-well plates, and Transwell chambers (purchased from Corning, USA) required for cell plating at -20°C in advance. We thawed the Matrigel (purchased from Corning, USA) at 4°C. Using serum-free medium, we diluted the Matrigel at 9 : 1, coated the upper chamber surface with it, and placed the plates at 37°C for 30 minutes to allow the Matrigel to polymerize. Before preparing the cell suspension, we treated the cells with starvation medium for 6 hours and then used trypsin to harvest the cells. Trypsin was neutralized, and cells were centrifuged to discard the culture solution, rinsed 1-2 times with PBS, and resuspended in serum-free medium containing bovine serum albumin. The cell density was adjusted to 5 × 105/ml. Aliquots of 100 *μ*l cell suspension were plated in the Transwell upper chambers. We then added 600 *μ*l of HUVEC complete medium to the lower chambers of each Transwell, taking care to avoid air bubbles during the seeding process. After 48 h incubation, we discarded the culture medium in the lower chamber, washed the Transwells twice with PBS, and fixed the cells with 4% formaldehyde for 30 minutes. We stained cell monolayers with 0.1% crystal violet solution for 20 minutes, washed with PBS three times, and gently wiped off unmigrated cells on the upper surface of the Transwell. After the Transwell had dried, we acquired micrographs and quantified cell migration through the Transwell.

### 2.8. Statistical Analysis

The data are expressed as the mean ± SD. The unpaired Student's *t* test was used to compare differences between the two groups. One-way analysis of variance was used to compare differences between more than two groups. *P* < 0.05 were considered statistically significant.

## 3. Results

### 3.1. DUSP22 Expression Level in TGF-*β* Induced EndMT

**Figure 1:**
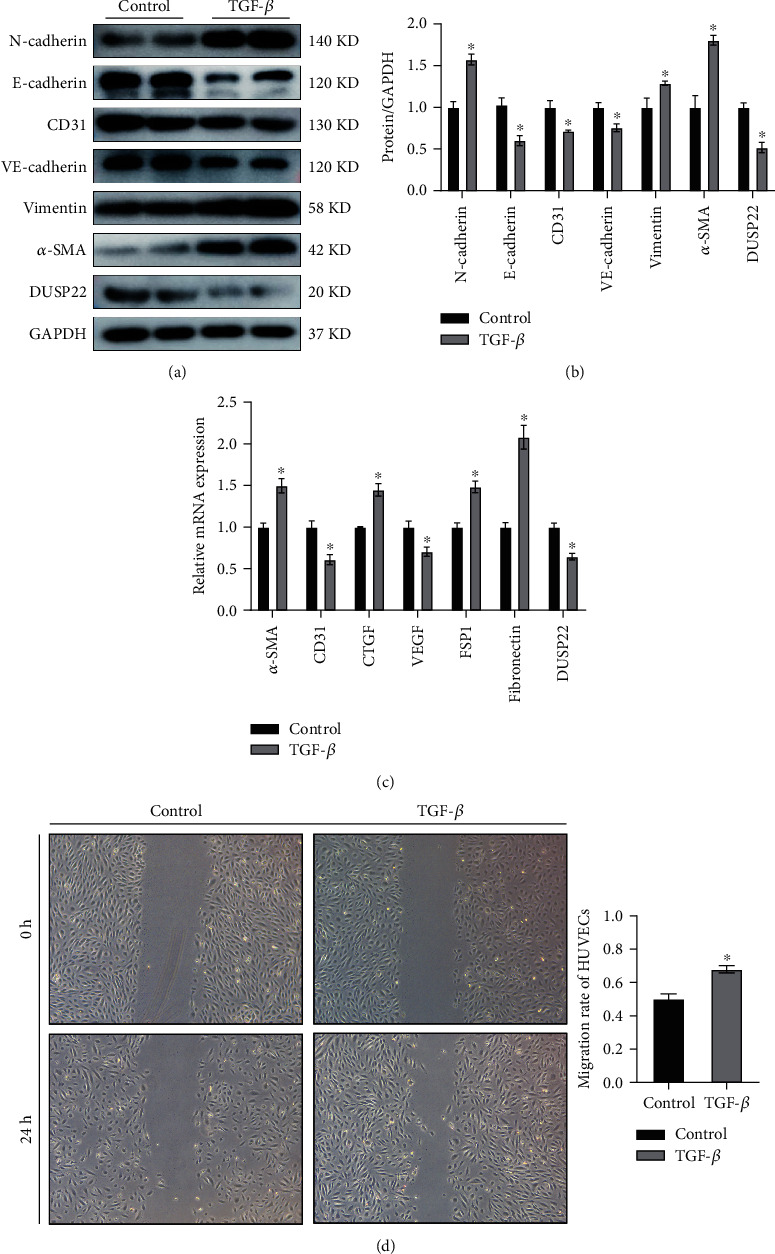
DUSP22 expression level in TGF-*β*-induced EndMT. DUSP22 expression after TGF-*β* (10 ng/ml) induced HUVECs 48 h indicated (a) representative blots and (b) quantitative results of the phenotypic molecule of the EndMT, (c) RT-PCR analysis, and (d) the cell migration. ^∗^*P* < 0.01 vs. control.

To determine whether DUSP22 participates in the TGF-*β*-induced EndMT process in HUVECs, we examined the mRNA and protein levels of DUSP22. By stimulating HUVECs 24 h and 48 h with TGF-*β* and taking the experience of previous experiments, we adopted 48 h stimulation to induce EndMT (Figure S1). After incubating HUVECs 48 h with 10 ng/ml TGF-*β*, we found that HUVECs go through the EndMT phenotypic change: the expression of endothelial cell markers including CD31, VE-cadherin, E-cadherin, and VEGF was downregulated, while the expression of mesenchymal cell markers including *α*-SMA, vimentin, N-cadherin, FSP1, and fibronectin was upregulated. Our data showed a decreasing trend in the mRNA and protein levels of DUSP22 after HUVECs had undergone EndMT (*P* < 0.01) (Figures 1(a)–1(c)). EndMT changed both protein levels and cell behavior. Therefore, we found that after TGF-*β* induction, the migration rate of HUVECs had been improved (Figure 1(d)). We conclude that DUSP22 may play a role in regulating EndMT.

### 3.2. DUSP22 Downregulation in HUVECs Aggravates EndMT and Cell Behavior

**Figure 2:**
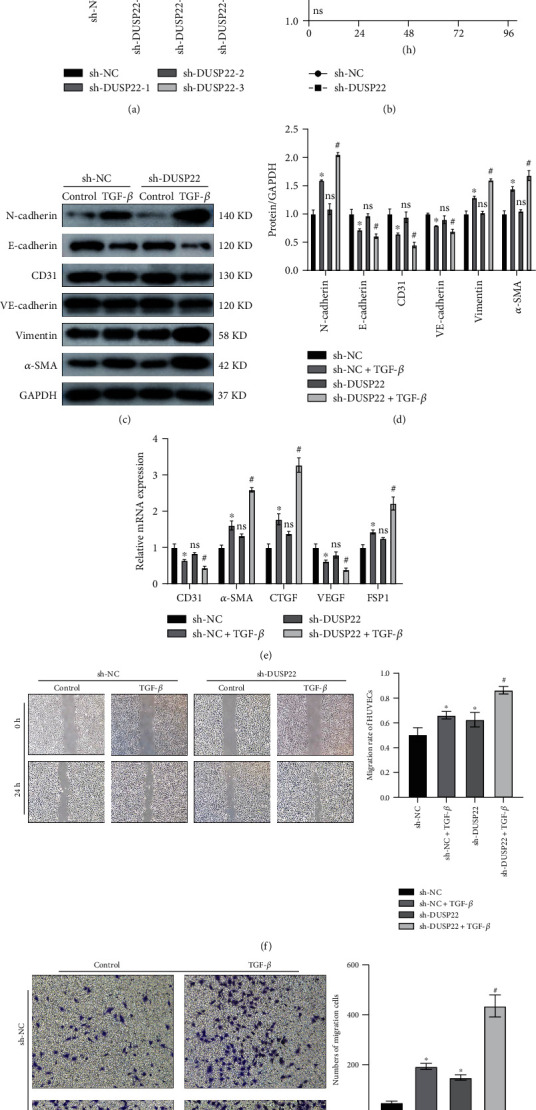
DUSP22 downregulation in HUVECs aggravates EndMT. (a) sh-DUSP22 reduced expression of DUSP22 in HUVECs; (b) the cell activity after transfecting the sh-NC and sh-DUSP22; the (c) protein representative blots and (d) quantitative results and the (e) mRNA expression changes of the phenotypic molecule of EndMT after transfecting the sh-NC and sh-DUSP22; the (f) cell migration and (g) invasion changes after transfecting the sh-NC and sh-DUSP22. ^∗^*P* < 0.01 vs. sh-NC. ^#^*P* < 0.01 vs. sh-NC+TGF-*β*.

We used short hairpin RNAs (shRNAs) sh-DUSP22-1, sh-DUSP22-2, and sh-DUSP22-3 to transfect HUVECs and quantify the shRNA-mediated knockdown efficiency of DUSP22 expression. Compared to the negative control shRNA (sh-NC) transfected group, the three experimental shRNA-transfected groups of cells showed significant reductions in DUSP22 expression (*P* < 0.01) (Figure 2(a)). Hence, we chose sh-DUSP22-1 for further experiments. After shRNA encoded viral transfection of HUVECs, the OD value in the sh-NC group was significantly higher than that in the sh-DUSP22 group at the 24 h, 48 h, 72 h, and 96 h time points (*P* < 0.01) (Figure 2(b)). The cell viability of the sh-NC-transfected group was significantly higher than that of the sh-DUSP22-transfected group. Western blot (WB) data showed that the protein expression levels of endothelial markers CD31, VE-cadherin, and E-cadherin in the sh-DUSP22+TGF-*β* group were significantly decreased and the protein expression levels of mesenchymal markers *α*-SMA, vimentin, and N-cadherin were significantly increased (*P* < 0.01) compared to the sh-NC+TGF-*β* group (Figures 2(c) and 2(d)). RT-PCR results showed that the mRNA expression levels of endothelial markers CD31 and VEGF were significantly decreased in the sh-DUSP22+TGF-*β* group and the mRNA expression levels of mesenchymal markers *α*-SMA, CTGF, FSP1, and fibronectin were significantly increased (*P* < 0.01) compared to the sh-NC+TGF-*β* group (Figure 2(e)). Twenty-four hours after making the monolayer cell scratch, cell migration rate in the sh-NC+TGF-*β* group was higher compared to the sh-NC group, and the scratch gap was narrower. Similarly, compared to the sh-NC+TGF-*β* group, cell migration in the sh-DUSP22+TGF-*β* group was higher, and the scratch gap was significantly narrower (*P* < 0.01) (Figure 2(f)). Our Transwell assay data showed that compared to the sh-NC group, the cells in the sh-NC+TGF-*β* group had a higher invasion ability and cells invaded in higher numbers (*P* < 0.01). Similarly, compared to the sh-NC+TGF-*β* group, the sh-DUSP22+TGF-*β* group showed stronger invasion ability and cells invaded in higher numbers (*P* < 0.01) (Figure 2(g)). We conclude that DUSP22 downregulation can aggravate EndMT and cell behavior.

### 3.3. DUSP22 Overexpression in HUVECs Ameliorates EndMT and Cell Behavior

**Figure 3:**
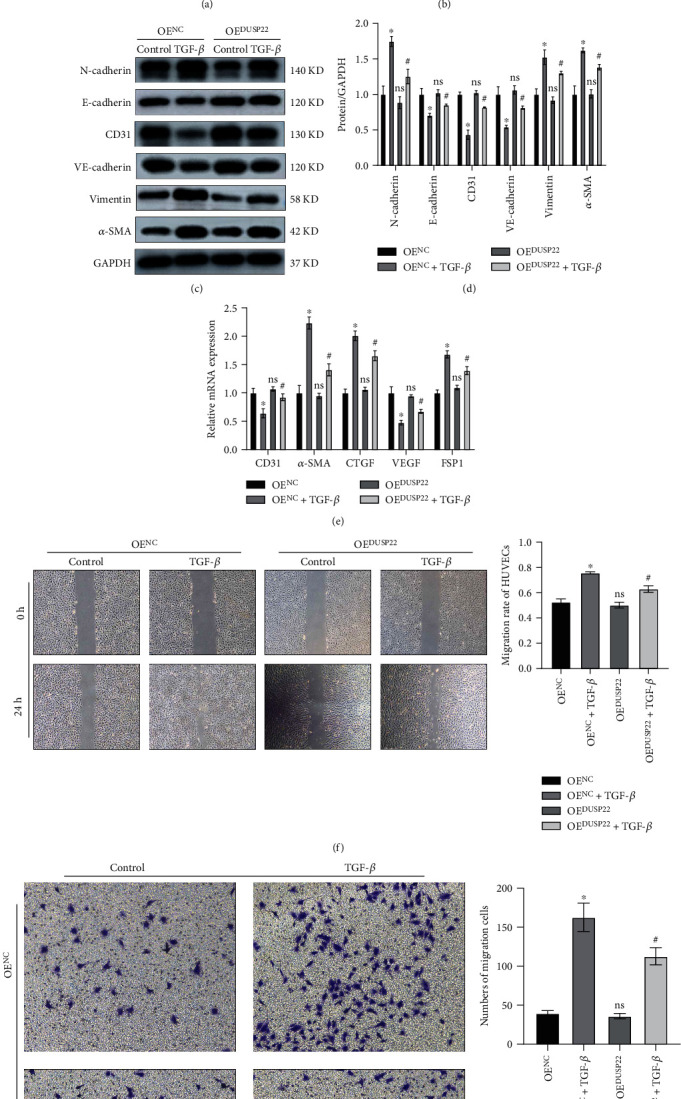
DUSP22 overexpression in HUVECs ameliorates EndMT. (a) OE^DUSP22^ reduced expression of DUSP22 in HUVECs; (b) the cell activity after transfecting the OE^NC^ and OE^DUSP22^; the (c) protein representative blots and (d) quantitative results and the (e) mRNA expression changes of the phenotypic molecule of EndMT after transfecting the OE^NC^ and OE^DUSP22^; the (f) cell migration and (g) invasion changes after transfecting the OE^NC^ and OE^DUSP22^. ^∗^*P* < 0.01 vs. OE^NC^. ^#^*P* < 0.01 vs. OE^NC^+TGF-*β*.

We used OE^DUSP22^ to transfect HUVECs to verify the OE^DUSP22^ overexpression efficiency. Compared with the OE^NC^ group, the cells in the OE^DUSP22^ have significantly increased DUSP22 gene expression (*P* < 0.01) (Figure 3(a)). After HUVECs were transfected with the virus, the OD value of the OE^NC^ group was significantly higher than that of the OE^DUSP22^ group at the 24 h, 48 h, 72 h, and 96 h time points (*P* < 0.01) (Figure 3(b)). Compared with the OE^NC^ group, the cells in the OE^DUSP22^ group had higher cell migration activity. After HUVECs in the OE^NC^ and OE^DUSP22^ groups were incubated with TGF-*β* (10 ng/ml) continuous stimulation for 48 h, compared with the OE^NC^+TGF-*β* group, WB results showed that the protein expression levels of CD31, VE-cadherin, and E-cadherin in the OE^DUSP22^+TGF-*β* group were significantly increased and the protein expression levels of *α*-SMA, vimentin, and N-cadherin were significantly decreased (*P* < 0.01) (Figures 3(c) and 3(d)). RT-PCR results showed that mRNA expression levels of CD31 and VEGF were significantly increased in the OE^DUSP22^+TGF-*β* group and the mRNA expression levels of *α*-SMA, CTGF, FSP1, and fibronectin were significantly decreased (*P* < 0.01) (Figure 3(e)). Twenty-four hours after making the cell scratch, cell migration rate was inactive, and the scratch gap was significantly wider in the OE^DUSP22^+TGF-*β* group (*P* < 0.01) compared to the OE^NC^+TGF-*β* group (Figure 3(f)). Our Transwell cell migration data showed that the cells had much weaker invasion ability and fewer numbers of cell invaded in the OE^DUSP22^+TGF-*β* group (*P* < 0.01) compared to the OE^NC^+TGF-*β* group (Figure 3(g)). We conclude that overexpression of DUSP22 can ameliorate EndMT and cell behavior.

### 3.4. DUSP22 Affects the smad2/3 Pathway

**Figure 4:**
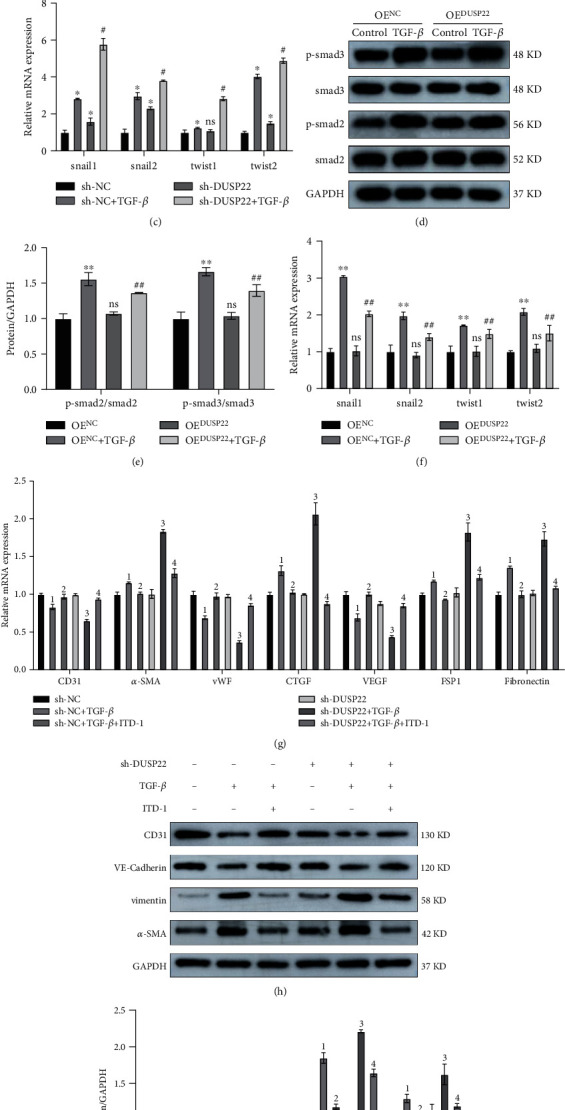
DUSP22 affects the smad2/3 signaling pathway. sh-DUSP22 activates the smad2/3 signaling pathway. The (a) protein representative blots and (b) quantitative results and the (c) mRNA expression changes of the downstream molecules of smad2/3 signaling pathway. ^∗^*P* < 0.01 vs. sh-NC. ^#^*P* < 0.01 vs. sh-NC+TGF-*β*. OE^DUSP22^ inactivates the smad2/3 signaling pathway. The (d) protein representative blots and (e) quantitative results and the (f) mRNA expression changes of the downstream molecules of smad2/3 signaling pathway. ^∗∗^*P* < 0.01 vs. OE^NC^. ^##^*P* < 0.01 vs. OE^NC^+TGF-*β*. After adding with smad2/3 inhibitor ITD-1, the (g) mRNA expression changes and the (h) protein representative blots and (i) quantitative results of the phenotypic molecule of EndMT. ^1^*P* < 0.01 vs. sh-NC. ^2^*P* < 0.01 vs. sh-NC+TGF-*β*. ^3^*P* < 0.01 vs. sh-DUSP22. ^4^*P* < 0.01 vs. sh-DUSP22+TGF-*β*.

The TGF-*β*/smad signaling pathway is the classic pathway of EndMT that can increase the expression of downstream signaling molecules. Hence, we chose to probe the smad2/3 signaling pathway to verify the role of DUSP22. After different interventions in the four groups of the knockdown model, WB results showed that compared to the sh-NC group, the protein expression of p-smad2/smad2 and p-smad3/smad3 in the sh-NC+TGF-*β* group was significantly upregulated (*P* < 0.01). Similarly, the above indicators were significantly upregulated in the sh-DUSP22+TGF-*β* group (*P* < 0.01) compared to the sh-NC+TGF-*β* group. There was no significant difference in the above indicators in the sh-DUSP22 group alone (*P* > 0.05) compared to the sh-NC group (Figures 4(a) and 4(b)). RT-PCR results showed that compared with the sh-NC group, the downstream molecules snail1, snail2, twist1, and twist2 of the EndMT signaling pathway in the sh-NC+TGF-*β* group were significantly upregulated (*P* < 0.01). Similarly, the downstream molecules in the sh-DUSP22+TGF-*β* group were significantly upregulated (*P* < 0.01) compared with the sh-NC+TGF-*β* group. There was no significant difference in the above indicators in the sh-DUSP22 group (*P* > 0.05) (Figure 4(c)).

After different interventions in the four groups of the overexpression model, WB results showed that the protein expression of p-smad2/smad2 and p-smad3/smad3 was significantly upregulated in the OE^NC^+TGF-*β* group (*P* < 0.01) compared to the OE^NC^ group. The above indicators in the OE^DUSP22^+TGF-*β* group were significantly downregulated (*P* < 0.01) compared with the OE^NC^+TGF-*β* group (Figures 4(d) and 4(e)). RT-PCR results showed that compared with the OE^NC^ group, the downstream molecules snail1, snail2, twist1, and twist2 in the OE^NC^+TGF-*β* group were significantly upregulated (*P* < 0.01). The downstream molecules were significantly downregulated (*P* < 0.01) compared with the OE^DUSP22^+TGF-*β* group (Figure 4(f)).

After the cells in the knockdown model were subjected to different interventions, we added the smad2/3 pathway small molecule inhibitor ITD-1 to verify the effect of DUSP22 on the changes of the smad2/3 signaling pathway. The RT-PCR results showed that compared with the sh-DUSP22+TGF-*β* group, the mRNA levels of endothelial cell phenotype molecules such as CD31, vWF, and VEGF were significantly upregulated, while the mRNA levels of mesenchymal phenotype molecules such as *α*-SMA, CTGF, fibronectin, and FSP1 were significantly downregulated in the EndMT model of HUVECs in the sh-DUSP22+TGF-*β*+ITD-1 group (*P* < 0.01) (Figure 4(g)). Our WB results showed that compared with the sh-DUSP22+TGF-*β* group, the protein expressions of CD31 and VE-cadherin were significantly upregulated and the protein expressions of *α*-SMA and vimentin were significantly downregulated in the sh-DUSP22+TGF-*β*+ITD-1 group (*P* < 0.01) (Figures 4(h) and 4(i)). We speculate that DUSP22 may ameliorate EndMT through dephosphorylating smad2/3.

### 3.5. DUSP22 Impacts the MAPK Pathway

**Figure 5:**
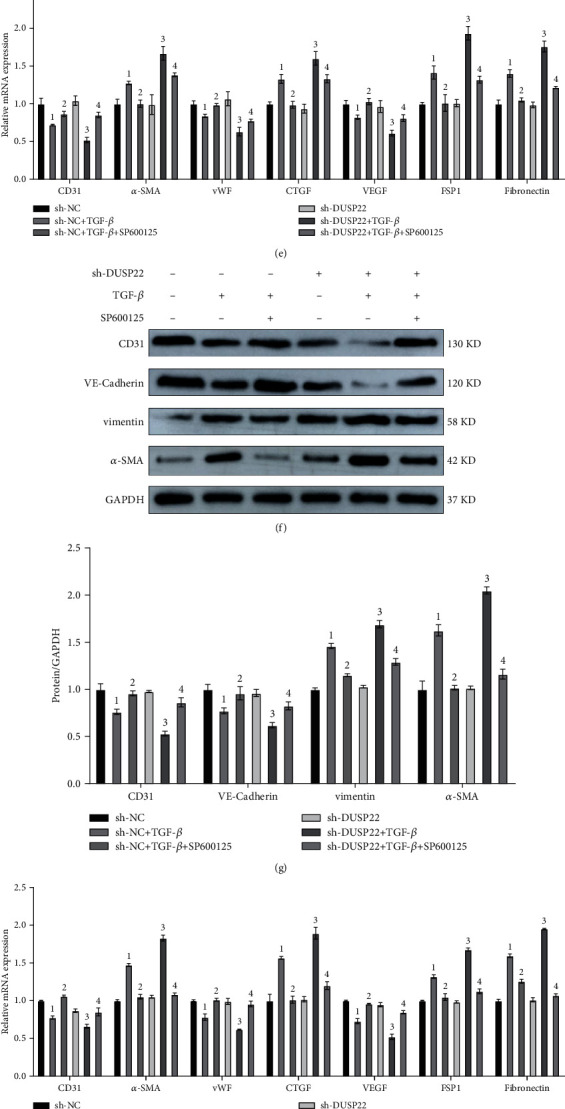
DUSP22 impacts the MAPK signaling pathway. sh-DUSP22 activates the MAPK signaling pathway. The (a) protein representative blots and (b) quantitative results of the p-JNK/JNK and p-ERK/ERK. ^∗^*P* < 0.01 vs. sh-NC. ^#^*P* < 0.01 vs. sh-NC+TGF-*β*. OE^DUSP22^ inactivates the MAPK signaling pathway. The (c) protein representative blots and (d) quantitative results of the p-JNK/JNK and p-ERK/ERK. ^∗∗^*P* < 0.01 vs. OE^NC^. ^##^*P* < 0.01 vs. OE^NC^+TGF-*β*. After adding with JNK inhibitor SP600125, the (e) mRNA expression changes and the (f) protein representative blots and (g) quantitative results of the phenotypic molecule of EndMT. After adding with ERK inhibitor ravoxertinib, the (h) mRNA expression changes and the (i) protein representative blots and (j) quantitative results of the phenotypic molecule of EndMT. ^1^*P* < 0.01 vs. sh-NC. ^2^*P* < 0.01 vs. sh-NC+TGF-*β*. ^3^*P* < 0.01 vs. sh-DUSP22. ^4^*P* < 0.01 vs. sh-DUSP22+TGF-*β*.

As an atypical DUSP, DUSP22 can regulate MAPK signaling transduction and is expressed in various types of tissues and cells. It was also known as JKAP and has been shown to dephosphorylate JNK and ERK in former studies. Therefore, we chose the MAPK signaling pathway to verify the role of DUSP22. By adding inhibitors of JNK and ERK, it was found that there was no effect on the smad2/3 pathway, and the interaction between the two pathways could be ruled out (Figure S2). After different interventions in the four groups of the knockdown model, WB results showed that compared with the sh-NC group, the protein expression of p-JNK/JNK and p-ERK/ERK in the sh-NC+TGF-*β* group was significantly upregulated (*P* < 0.01). Also, compared with the sh-NC+TGF-*β* group, the above indicators were more significantly upregulated in the sh-DUSP22+TGF-*β* group (*P* < 0.01). Finally, compared with the sh-NC group, there was no significant difference in the above indicators in the sh-DUSP22 group alone (*P* > 0.05) (Figures 5(a) and 5(b)).

After different interventions in the four groups of the overexpression model, WB results showed that compared with the OE^NC^ group, the protein expression of p-JNK/JNK and p-ERK/ERK was significantly upregulated in the OE^NC^+TGF-*β* group (*P* < 0.01). Also, compared with the OE^NC^+TGF-*β* group, the above indicators in the OE^DUSP22^+TGF-*β* group were significantly downregulated (*P* < 0.01) (Figures 5(c) and 5(d)).

After cells in the knockdown model were subjected to different interventions, we chose JNK inhibitor SP600125 and ERK inhibitor ravoxertinib to probe the impact of DUSP22 on changes in the MAPK signaling pathway. Our RT-PCR results showed that the mRNA expressions of CD31, vWF, and VEGF were significantly upregulated compared to the sh-DUSP22+TGF-*β* group. Also, the mRNA expressions of *α*-SMA, CTGF, fibronectin, and FSP1 were significantly downregulated in the sh-DUSP22+TGF-*β*+SP600125 group (*P* < 0.01) (Figure 5(e)) and the sh-DUSP22+TGF-*β*+ravoxertinib group (*P* < 0.01) (Figure 5(h)). Our WB results showed that the protein expression of CD31 and VE-cadherin was significantly upregulated and the protein expression of *α*-SMA and vimentin was significantly downregulated in the sh-DUSP22+TGF-*β*+SP600125 group (*P* < 0.01) (Figures 5(f) and 5(g)) and sh-DUSP22+TGF-*β*+ravoxertinib group (*P* < 0.01) compared to the sh-DUSP22+TGF-*β* group (Figures 5(i) and 5(j)). We speculated that DUSP22 may ameliorate EndMT through dephosphorylating JNK and ERK.

## 4. Discussion

Cardiac fibrosis is the expansion of the cardiac interstitium caused by the net accumulation of ECM protein, and this is accompanied by numerous cardiac pathological conditions. In most cardiac diseases, the degree of cardiac fibrosis indicates adverse consequences. There are some growth factors that can stimulate EndMT including TGF-*β*, BMPs (bone morphogenetic proteins), hypoxia, inflammatory cytokines (TNF*α*, IL-1*β*), and hemodynamic shear stress. In view of previous studies and some limits, we only used TGF-*β* to induce HUVECs to construct the EndMT model. TGF-*β* is the most typical fibrogenic growth factor [37, 38]. Several studies support a role for TGF-*β* in the pathogenesis of cardiac fibrosis. There are many cardiovascular phenomena induced by TGF-*β*. If the endothelial cell phenomenon induced by HUVEC is used *in vitro*, it can simulate atherosclerosis (mainly due to endothelial cell damage) and myocardial fibrosis (mainly due to the conversion of endothelial cells into mesenchymal cells) in clinical models [39–41]. TGF-*β* stimulation can effectively induce myofibroblast transformation and increase ECM protein synthesis of activated fibroblasts [42]. The regulatory role and the importance of TGF-*β* in signaling pathways of cardiac and vascular morphogenesis and development and cardiovascular homeostasis are obvious. For example, TGF-*β*-deficient mice show age-related reduction in myocardial fibrosis and improved cardiac compliance. In contrast, TGF-*β*-overexpressed mice had significant ventricular fibrosis and increased numbers of cardiac fibroblasts [43]. Therefore, in our experiments, we chose TGF-*β* to induce HUVECs in an *in vitro* EndMT model.

DUSP22, also known as JKAP, inactivates MAPK and other tyrosine kinases through dephosphorylation. DUSP22 can exert its physiological functions in many ways. (1) DUSP22 inhibits the MAPK pathway by dephosphorylating JNK, ERK, and p38 [23, 44]. (2) DUSP22 participates in the ASK1-MKKK7-JNK signal transduction pathway and mediates cell apoptosis [45]. (3) DUSP22 participates in inflammatory diseases and reduces the production of inflammatory cytokines [20, 45, 46]. (4) DUSP22 inhibits the STAT3 signal pathway activated by IL-6 [35, 46]. (5) DUSP22 dephosphorylates FAK and supports cell migration [47]. At present, there are no published reports focused on the role of DUSP22 in cardiovascular diseases. Due to the broad impact of DUSP22 on MAPK activation and inflammation- and fibrosis-associated diseases, we hypothesized that there may be a potential functional involvement of DUSP22 in EndMT. This line of reasoning led us to the hypothesis that DUSP22 may play a regulatory role in cardiovascular diseases through these signaling pathways. Hence, we chose DUSP22 to verify its function in EndMT.

Using our *in vitro* model of EndMT, we found that a 48 h TGF-*β* stimulation of HUVECs decreased the expression of endothelial markers CD31, VE-cadherin, E-cadherin, and VEGF and increased the expression of mesenchymal markers *α*-SMA, vimentin, N-cadherin, CTGF, fibronectin, and FSP1, indicating that our EndMT model was responding appropriately and was successfully constructed. DUSP22 expression in our EndMT model decreased significantly, indicating that DUSP22 is likely to participate in the EndMT and may play an inhibitory role. Therefore, we have reason to suspect that the DUSP22 gene may be involved in the TGF-*β*-induced EndMT process in HUVECs.

Studies have shown that DUSP22 colocalizes with actin filaments at the plate foot at the leading edge of migrating cells [33], suggesting that DUSP22 can regulate cell motility. Additionally, cells expressing DUSP22 migrated significantly slower, whereas expression of DUSP22 phosphatase mutant (DUSP22-C88S) and downregulation of endogenous DUSP22 significantly enhanced growth factor-induced cell migration [48]. Increased expression of DUSP22 significantly decreased FAK phosphorylation, whereas downregulation of DUSP22 enhanced cell migration and FAK phosphorylation. By modulating the expression and activity of DUSP22, a novel role of this phosphatase in coordinating cell motility by regulating FAK phosphorylation has been demonstrated [47]. These studies showed that DUSP22 can regulate cell migration through multiple signaling pathways.

In our data, decreased expression of DUSP22 promoted while increased expression of DUSP22 inhibited the phenotypic conversion process of EndMT. From a histological point of view, during EndMT, the integrity of the endothelial cells is disrupted, exhibiting migratory, invasive, and proliferative phenotypes [49]. Our results show that TGF-*β* induced EndMT in HUVECs and increased their cell migration and invasion abilities. Furthermore, we found that reducing DUSP22 expression enhanced the ability of cell migration and invasion whereas increasing DUSP22 expression weakened the ability of cell migration and invasion.

TGF-*β*/smad signaling pathway is the classic pathway of EndMT [50, 51] that can increase the expression of downstream signaling molecules such as snail1, snail2, twist1, and twist2 [52]. Our results show that TGF-*β* induction reduced DUSP22 expression and significantly increased the expression levels of downstream signaling proteins. Increased DUSP22 expression led to a downregulation of downstream signaling proteins. TGF-*β* has been shown to increase the expression of p-smad2 and p-smad3. Our data also confirm this: the expression of p-smad2 and p-smad3 was significantly increased after TGF-*β* induction. Reducing DUSP22 expression promotes phosphorylation of smad2 and smad3, while increasing DUSP22 expression inhibits phosphorylation of smad2 and smad3. We experimentally inhibited the phosphorylation of smad2 and smad3 by adding inhibitor ITD-1 [53] and speculate from these data that DUSP22 may regulate EndMT by inhibiting the phosphorylation of smad2 and smad3.

JNK/MAPK and ERK/MAPK signaling pathways can play important roles in various cardiovascular diseases [54, 55]. Although there are few studies on the changes of JNK and ERK in the process of EndMT, DUSP22 can regulate and affect JNK/MAPK and ERK/MAPK pathways. We investigated whether reducing or increasing the expression of DUSP22 would affect the expression and phosphorylation of JNK and ERK during EndMT. Our results revealed that knocking down DUSP22 expression led to a decrease in JNK expression [44], a corresponding decrease in p-JNK expression and decrease in p-ERK expression. However, TGF-*β*-induced HUVECs still showed an increase in JNK expression, a corresponding p-JNK expression increase and a p-ERK expression increase. We speculate that DUSP22 downregulation may promote the phosphorylation of JNK and ERK in the TGF-*β*-induced EndMT process. Since DUSP22 can inactivate JNK/MAPK pathway-related factors through the dephosphorylation of serine/threonine residues, overexpression of DUSP22 may inhibit the phosphorylation of JNK and ERK in TGF-*β*-induced EndMT. In the context of reducing or overexpressing DUSP22, the expression changes of p38 and p-p38 have no corresponding relationship with the expression of DUSP22. We reason that DUSP22 may not affect the process of EndMT by affecting the phosphorylation of p38. Additionally, we further verified that DUSP22 affects the MAPK signaling pathway by using JNK inhibitor SP600125 [56] and ERK inhibitor ravoxertinib [57] in our experiments. From the results of these additional experiments, we speculate that DUSP22 expression may dephosphorylate JNK and ERK to inhibit activation of the MAPK signaling pathway.

## 5. Conclusion

**Figure 6:**
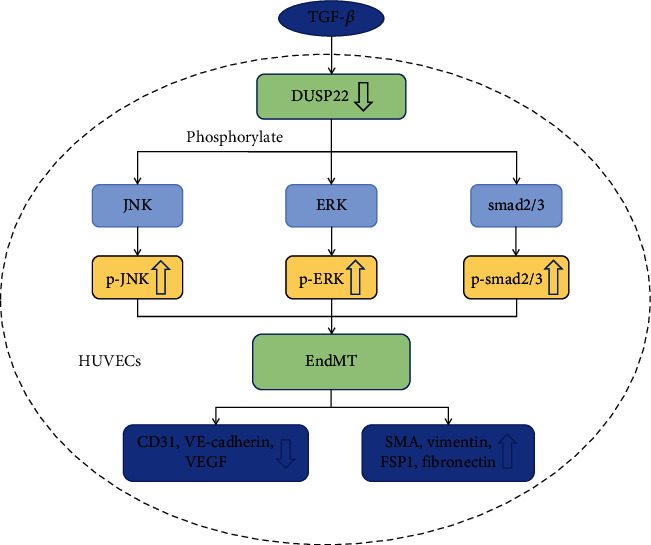
The signaling pathways of DUSP22 in EndMT. During TGF-*β*-induced EndMT, the expression of DUSP22 is downregulated. This decrease in DUSP22 expression level aggravated EndMT, while the overexpression of DUSP22 ameliorated EndMT in HUVECs. DUPS22 affects EndMT through smad2/3 and MAPK signaling pathways.

In conclusion, our study demonstrated that during TGF-*β*-induced EndMT, the expression of DUSP22 is downregulated. This decrease in DUSP22 expression level aggravated EndMT, while the overexpression of DUSP22 ameliorated EndMT in HUVECs. DUPS22 affects EndMT through smad2/3 and MAPK signaling pathways (Figure 6). Thus, targeting DUSP22 may become a new therapeutic strategy for inhibiting the progression of cardiac remodeling by regulating EndMT.

However, to provide a more comprehensive understanding of how DUSP22 regulates EndMT, we could consider using some of these other stimuli either alone or in combination with TGF-*β* in future studies and explore some other signaling pathways that could potentially play a role including PI3K/AKT, Wnt/*β*-catenin, and NF-*κ*B. Moreover, we did not perform *in vivo* experiments to validate our *in vitro* results. Hence, we cannot confirm whether DUSP22 can play a role in regulating EndMT in living animals. Thinking about the conclusion suggesting a role for DUSP22 in targeting strategies, we have not found studies of DUSP22 in cardiovascular clinical researches, so we cannot accurately conclude the relationship between DUSP22 in healthy people and patients with cardiovascular disease. Therefore, our next experiment can collect samples from clinical patients to study the changes in DUSP22 expression.

## Figures and Tables

**Table 1:** Primer sequences.

Primer	5′ to 3′
*β*-Actin	F: GGTCATCACCATTGGCAA
	R: GAGTTGAAGGTAGTTTCGTGGA

CD31	F: CACTTCTGAACTCCAACAACG
	R: GGACACTTGAACTTCCGTG

*α*-SMA	F: TGAAGAGCATCCCACCCT
	R: ATAGAGAGACAGCACCGCC

FSP1	F: CAAAGAGGGTGACAAGTTCAAG
	R: ACAGGAAGACACAGTACTCTTG

CTGF	F: ATTCTGTGGAGTATGTACCGAC
	R: GTCTCCGTACATCTTCCTGTAG

VEGF	F: ATCGAGTACATCTTCAAGCCAT
	R: GTGAGGTTTGATCCGCATAATC

Fibronectin	F: AATAGATGCAACGATCAGGACA
	R: GCAGGTTTCCTCGATTATCCTT

DUSP22	F: AGTTAAATACCTGTGCATCCCA
	R: CGGCACTCGTGAATGAATTTAA

vWF	F: CCTGTTACTATGACGGTGAGAT
	R: CATGAAGCCATCCTCACAGTAG

snail1	F: CCTCGCTGCCAATGCTCATCTG
	R: AGCCTTTCCCACTGTCCTCATCTG

snail2	F: CTGTGACAAGGAATATGTGAGC
	R: CTAATGTGTCCTTGAAGCAACC

twist1	F: CCTCGGACAAGCTGAGCAAGATTC
	R: GTCGCTCTGGAGGACCTGGTAG

twist2	F: GCAGAGCGACGAGATGGACAATAAG
	R: CTAGTGGGAGGCGGACATGGAC

## Data Availability

The data used to support the findings of this study are available from the corresponding author upon request.

## Supplementary Materials

Supplementary Materials The supplementary figure descriptions: Figure S1: DUSP22 expression level in TGF-*β*-induced EndMT. Figure S2: the effects of JNK and ERK inhibitors on the pSMAD levels.
