# Supplementary material for: DUSP22 Ameliorates Endothelial-to-Mesenchymal Transition in HUVECs through Smad2/3 and MAPK Signaling Pathways
Source: Cardiovasc Ther. 2024 Mar 8;2024:5583961. doi: 10.1155/2024/5583961 (PMC10942825; doi:10.1155/2024/5583961)
Supplement: Supplementary Materials — The supplementary figure descriptions: Figure S1: DUSP22 expression level in TGF-β-induced EndMT. Figure S2: the effects of JNK and ERK inhibitors on the pSMAD levels. [file 5583961.f1.docx]

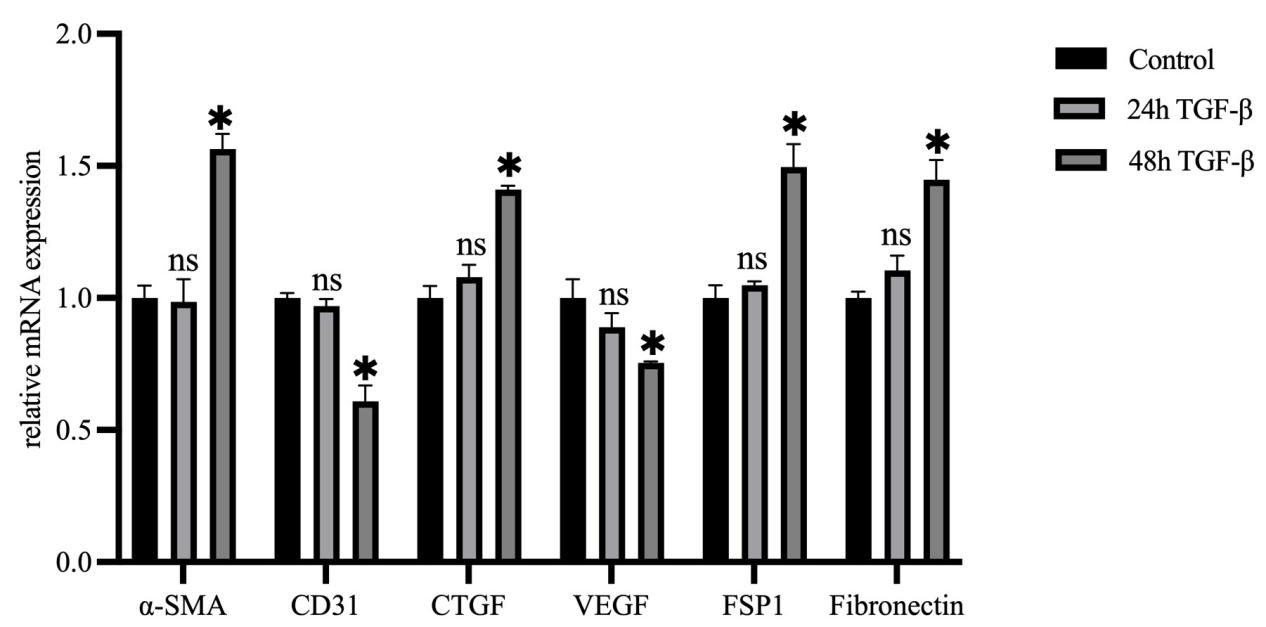


**Figure S1** **DUSP22 expression level in TGF-β induced EndMT.** The quantitative results of the phenotypic molecular of the EndMT TGF-β (10ng/ml) induced HUVECs 24h and 48h indicated in RT-PCR analysis. ^*^*P*<0.01 vs Control.


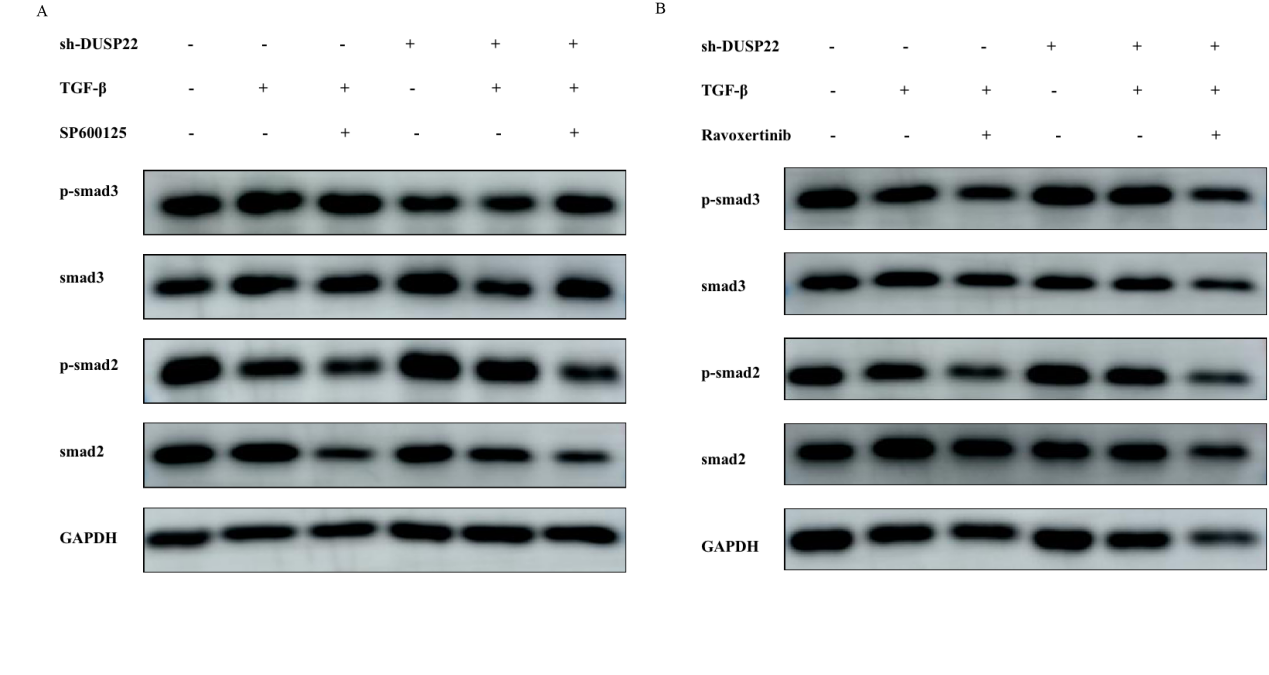


**Figure S2 The effects of JNK and ERK inhibitors on the pSMAD levels**. After adding inhibitors of JNK (A) and ERK (B), the WB results of the smad2/3 pathway.
